# Leveraging Large Language Models for Infectious Disease Surveillance—Using a Web Service for Monitoring COVID-19 Patterns From Self-Reporting Tweets: Content Analysis

**DOI:** 10.2196/63190

**Published:** 2025-02-20

**Authors:** Jiacheng Xie, Ziyang Zhang, Shuai Zeng, Joel Hilliard, Guanghui An, Xiaoting Tang, Lei Jiang, Yang Yu, Xiufeng Wan, Dong Xu

**Affiliations:** 1 Department of Electrical Engineering and Computer Science University of Missouri Columbia, MO United States; 2 Christopher S. Bond Life Sciences Center University of Missouri Columbia, MO United States; 3 School of Acupuncture-Moxibustion and Tuina Shanghai University of Traditional Chinese Medicine Shanghai China; 4 Shanghai Pudong New Area Wanggang Community Health Service Center Shanghai China; 5 NextGen Center for Influenza and Emerging Infectious Diseases University of Missouri Columbia United States; 6 Department of Molecular Microbiology and Immunology University of Missouri Columbia United States

**Keywords:** COVID-19, self-reporting data, large language model, Twitter, social media analysis, natural language processing, machine learning

## Abstract

**Background:**

The emergence of new SARS-CoV-2 variants, the resulting reinfections, and post–COVID-19 condition continue to impact many people’s lives. Tracking websites like the one at Johns Hopkins University no longer report the daily confirmed cases, posing challenges to accurately determine the true extent of infections. Many COVID-19 cases with mild symptoms are self-assessed at home and reported on social media, which provides an opportunity to monitor and understand the progression and evolving trends of the disease.

**Objective:**

We aim to build a publicly available database of COVID-19–related tweets and extracted information about symptoms and recovery cycles from self-reported tweets. We have presented the results of our analysis of infection, reinfection, recovery, and long-term effects of COVID-19 on a visualization website that refreshes data on a weekly basis.

**Methods:**

We used Twitter (subsequently rebranded as X) to collect COVID-19–related data, from which 9 native English-speaking annotators annotated a training dataset of COVID-19–positive self-reporters. We then used large language models to identify positive self-reporters from other unannotated tweets. We used the Hibert transform to calculate the lead of the prediction curve ahead of the reported curve. Finally, we presented our findings on symptoms, recovery, reinfections, and long-term effects of COVID-19 on the Covlab website.

**Results:**

We collected 7.3 million tweets related to COVID-19 between January 1, 2020, and April 1, 2024, including 262,278 self-reported cases. The predicted number of infection cases by our model is 7.63 days ahead of the official report. In addition to common symptoms, we identified some symptoms that were not included in the list from the US Centers for Disease Control and Prevention, such as lethargy and hallucinations. Repeat infections were commonly occurring, with rates of second and third infections at 7.49% (19,644/262,278) and 1.37% (3593/262,278), respectively, whereas 0.45% (1180/262,278) also reported that they had been infected >5 times. We identified 723 individuals who shared detailed recovery experiences through tweets, indicating a substantially reduction in recovery time over the years. Specifically, the average recovery period decreased from around 30 days in 2020 to approximately 12 days in 2023. In addition, geographic information collected from confirmed individuals indicates that the temporal patterns of confirmed cases in states such as California and Texas closely mirror the overall trajectory observed across the United States.

**Conclusions:**

Although with some biases and limitations, self-reported tweet data serves as a valuable complement to clinical data, especially in the postpandemic era dominated by mild cases. Our web-based analytic platform can play a significant role in continuously tracking COVID-19, finding new uncommon symptoms, detecting and monitoring the manifestation of long-term effects, and providing necessary insights to the public and decision-makers.

## Introduction

### Background

COVID-19 is one of the most severe infectious diseases in human history. Although the World Health Organization downgraded the COVID-19 pandemic, declaring it is no longer a global emergency on May 5, 2023 [1], the threat of infection and death remains. As of August 1, 2023, there have been >300,000 confirmed cases weekly worldwide, resulting in >1000 deaths per week; however, major information-publishing platforms, such as that at Johns Hopkins University, stopped collecting and tracking COVID-19 data worldwide on March 10, 2023 [2]. Therefore, it has become more challenging to identify the actual number and trends of COVID-19 infections daily. Traditional public health monitoring methods face several challenges, including delays in clinical data collection, lack of real-time insights, and the underrepresentation of population-level trends, particularly in regions with limited health care reporting infrastructure [3,4]. New tools are required to provide timely awareness and detection of COVID-19 transmission trends, reinfection patterns, and the long-term health impact of the disease.

To supplement the shortage of clinical data and gain further insights into the development trends and variant tendencies of COVID-19, researchers have turned to social media, specifically Twitter (subsequently rebranded as X). Social media data offer unique advantages, such as rapid updates and a broad geographical reach, which traditional clinical data often lack [5,6]. Several studies [7-11] have explored the use of social media for health monitoring, including sentiment analysis of COVID-19–related tweets, the identification of emerging symptoms, and the study of vaccine hesitancy. Early studies [12-16] primarily focused on constructing COVID-19–related tweet databases. However, these works do not provide in-depth analysis of self-reported tweets, and the databases tended to be collected over a short time frame, typically several months, and cannot automatically update. Later, some research endeavors [9,17-19] shifted their focus toward studying COVID-19 symptoms on the basis of tweets and reported the distribution of symptoms in tweets. However, these studies included limited numbers of collected self-reported cases. Some studies [16,20-24] used tweets to study geographic distribution but did not provide a corresponding time-series analysis or predict the spread of COVID-19. Moreover, existing studies have not fully leveraged user-generated content on social media to provide a comprehensive, dynamic view of COVID-19 trends across symptoms, geography, and time. This gap underscores the need for a methodology capable of providing near–real-time insights and broader geographical coverage [25]. As for the visualization tools for COVID-19, some researchers [26-29] have developed platforms or dashboards to study the trends of COVID-19, but most were based on clinical data. A few tweet-based platforms [27,29,30] showed limited information and failed to provide timely updates.

Reinfection often refers to the phenomenon in which an individual who has recovered from COVID-19 is again infected with the virus [31]. Some researchers [32,33] considered that reinfection is identified when an individual tests positive again through polymerase chain reaction (PCR) testing after a minimum of 90 days of a negative result. However, some studies also suggest this duration should be 30 days [34,35]. Other studies [34,36,37] considered reinfection to be a new positive PCR following 2 consecutive negative PCR tests taken after primary infection. Moreover, the periodicity of reinfection, the reinfection rate, and the maximum number of infections are uncertain. Existing studies on reinfection trends and periodicity have primarily relied on clinical data, leaving gaps in understanding population-level reinfection dynamics that may be observable through self-reported data on social media [38]. In addition to reinfection, there is a growing concern about the long-term effects of COVID-19, known as post–COVID19 condition, where patients report symptoms lasting months after recovery [39,40]. Clinical data on long-term effects remain limited [41], but social media offers a platform where individuals can share ongoing symptoms, providing valuable insights for public health research [42,43]. This further highlights the potential of using self-reported data to investigate the periodicity of reinfection, as well as long-term effects and symptoms, complementing traditional clinical datasets.

Health natural language processing (NLP) is gaining increasing attention for its essential role in both methodology development and applications [44]. The technology has been widely applied in areas such as information extraction from electronic health records [45], adverse drug reaction analysis [46], clinical decision support [47], and hospitalization prediction [48]. Using techniques such as topic modeling, sentiment analysis, and deep learning models such as Bidirectional Encoder Representations from Transformers (BERT) [49], NLP can extract valuable medical insights from unstructured text. Combined with real-time analytics frameworks [50] and knowledge graphs [51], these technologies can dynamically monitor COVID-19 trends, identify previously unnoticed symptoms and long-term effects, and provide scientific support for optimizing public health strategies and resource allocation.

### Objectives

**Figure 1:**
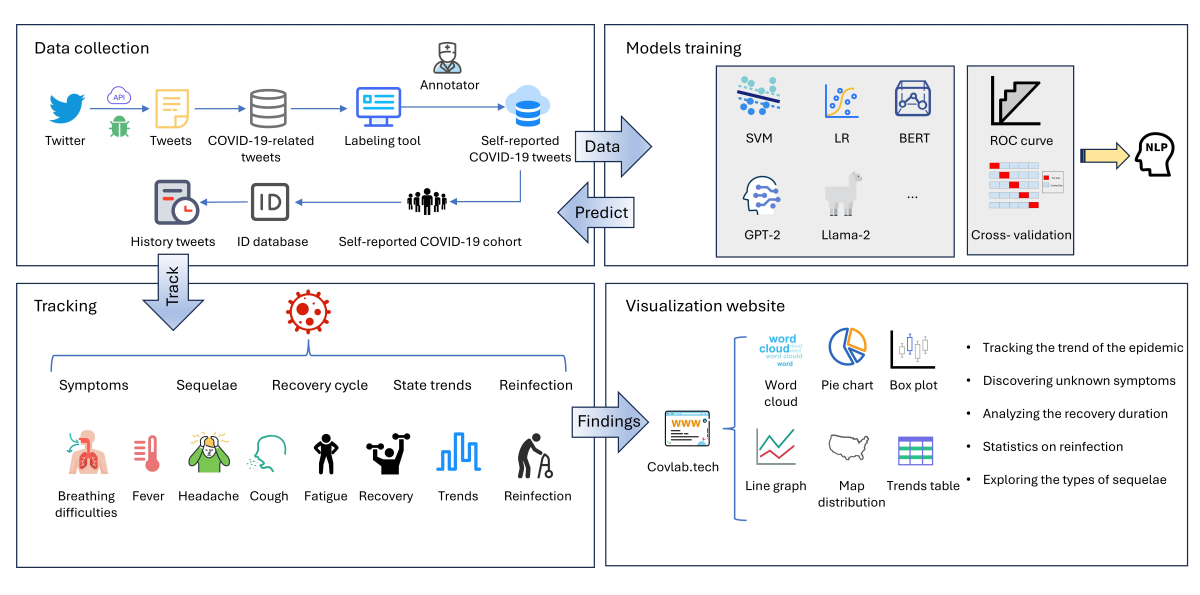
Workflow for Covlab. BERT: Bidirectional Encoder Representations from Transformers; Llama-2: large language model meta AI 2; LR: logistic regression; NLP: natural language processing; ROC: receiver operating characteristics; SVM: support vector machine.

In this study, we address these gaps by leveraging self-reported COVID-19 data from Twitter to provide near–real-time insights into trends across symptoms, reinfection, recovery cycles, and geographical distributions. The proposed approach not only fills the limitations of traditional monitoring systems but also offers a scalable, timely, and comprehensive method to track the pandemic’s evolving dynamics. Our visualization tool updates weekly and comprehensively analyzes information related to COVID-19 symptoms, case distribution, reinfections, recoveries, and long-term effects based on large language models (LLMs). Figure 1 depicts our research objectives: (1) we aim to build a publicly available database of >9,836,206 COVID-19–related tweets, and this database is set to automatically update with newly collected data weekly; (2) LLMs will be built to automatically filter the tweets of self-reporters and extract their mentioned symptoms and recovery cycles; and (3) we aim to build a visualization website that refreshes data on a weekly basis, Covlab [52], to track and analyze infection, reinfection, recovery, and long-term effects of COVID-19.

As depicted in Figure 1, the workflow of Covlab comprised the following steps: data collection, models training, tracking, and visualization website. During data collection, COVID-19–related tweets were collected using the Twitter application programming interface (API) and filtered from a broader dataset of social media posts. A subset of these tweets was manually labeled through a specialized annotation tool, creating a high-quality training set essential for developing machine learning models. In addition, historical tweets and user ID databases were incorporated to construct a comprehensive cohort of individuals who self-reported COVID-19 infections, forming the foundational dataset for subsequent tracking and analytical tasks. In models training step, annotated datasets were used to train a variety of machine learning and NLP models, including support vector machine (SVM), logistic regression (LR), BERT, GPT-2, and LLM meta AI 2 (Llama-2), to identify the most effective model for detecting self-reported COVID-19 infection tweets. After selecting the optimal model, it was applied to a larger collection of COVID-19–related tweets to extract tweets reporting personal COVID-19 infections. Throughout this process, performance metrics such as cross-validation and receiver operating characteristic (ROC) curve analysis, were used to ensure the models provided robust and accurate predictions. In the tracking step, the system conducted long-term tracking of individuals who self-reported COVID-19 infections in their tweets, focusing on analyzing symptoms such as breathing difficulties, fever, headache, cough, and fatigue, as well as recovery cycles, long-term effects (ie, sequelae), and geographical trends. In addition, reinfection cases were monitored to provide insights into the temporal dynamics of COVID-19 experiences, helping to uncover patterns in symptom progression, recovery, and recurring infections over time. In the visualization website step, the results of the analyses were presented on an interactive platform, Covlab, which provided a variety of visualization tools, including word clouds, pie charts, box plots, line graphs, geographic maps, and trend tables. This platform enabled users to track epidemic trends, uncover new or previously unreported symptoms, analyze recovery durations, report reinfection statistics, and explore different types of long-term effects (ie, sequelae), offering valuable insights to researchers and public health officials.

## Methods

### Data Collection and Preprocessing

We collected and processed tweet data from January 2020 to April 2024 based on COVID-19–related keywords and hashtags through the Twitter streaming API. The searching keywords included “I.* tested[ed] positive for [covid | coronavirus | covid19 | covid-19],” “My.* [covid | coronavirus | covid19 | covid-19].* symptoms,” “#COVID” “#LongCovid” (all the keywords can be found in Multimedia Appendix 1). The following preprocessing operations were conducted on the tweets collected based on keywords. We first converted all words in the tweets to lowercase, standardized the tweets to American Standard Code for Information Interchange encoding using the Unicode library, and tokenized the tweets. Next, we removed all Unicode symbols and punctuation marks, some uninformative characters about usernames, such as @username, all digits and line breaks in the tweets, all URLs, such as http://, and all words contained in our stop word library (Multimedia Appendix 2). We converted emoji expressions into their corresponding textual expressions. To provide sufficient datasets for subsequent model training, we constructed a self-reported COVID-19 tweets dataset with manual labeling by 9 native English-speaking annotators after obtaining ethics approval. We also established a set of calibration criteria as shown in Table 1 to ensure the consistency and reliability of the tweet annotations. Two additional annotators conducted a secondary annotation on the labeled data. If their new annotations were inconsistent with the originals, all annotators would decide through a voting process whether the tweet should be classified as a self-reported COVID-19 positive tweet. The Fleiss κ index [53] was used to measure the consistency among multiple annotators to ensure the reliability of the annotations (Multimedia Appendix 3). We also developed a web-based annotation tool (Multimedia Appendix 4) to improve the efficiency of manual annotation. We annotated 115,214 tweets, of which 13,701 (11.89%) were positive samples that described self-reported positive COVID-19 cases and 101,513 (88.11%) were negative samples either not describing self-infection with COVID-19 or irrelevant. Multimedia Appendix 5 presents the types of tweets targeted in our study. The tweet depicted on the left serves as a prototypical instance, delineating self-reported information about COVID-19 infections. This includes the date of diagnosis and a detailed account of the symptoms experienced by the individual. Conversely, although the tweet shown on the right also references a diagnosis of COVID-19 and details associated symptoms, it diverges from our criteria for target tweets because it recounts a diagnosis pertaining to a third party, specifically a friend of the tweeter, rather than a self-reported account. Therefore, it falls outside the scope of our target dataset.

**Table 1:** Labeling criteria for the tweets.

Index	Annotation guideline	Description
1	Self-reported infection	Tweets must be a personal account by the author regarding their experience of contracting COVID-19. If the tweet mentions someone other than the author being infected, such as friends, family, or others, it should be labeled as a negative sample.
2	First-person narrative	Tweets should use first-person pronouns (eg, “I,” “me,” and “my”) to describe the author’s experience of contracting COVID-19, not that of others.
3	Concrete information	Tweets should provide concrete details, including infection timeline, test results and medical treatments, among others, rather than general discussions or speculations.
4	Symptom description	Tweets should contain the patient’s personal descriptions of COVID-19 symptoms experienced, such as fever, cough, or difficulty breathing.
5	Confirmation information	Tweets should mention how the author was confirmed to have contracted COVID-19, such as the type of test conducted (PCR^a^ and rapid antigen test), confirmation by a physician, or official institution validation.
6	Treatment experience	Tweets should describe the author’s experience of treatment or recovery after self-contracting COVID-19, including isolation, medication, deterioration, or improvement in their health.
7	Infection timeline	Tweets should contain exact time points or time ranges of the infection rather than general discussions. Providing precise timing helps verify the author’s infection period.
8	Test results	Tweets should reference the author’s COVID-19 test results, such as testing positive or negative or other relevant test outcomes.
9	Medical Facility	Tweets should mention whether the author received treatment or underwent COVID-19-related tests at a medical facility, such as a hospital or clinic.
10	Social distancing measures	Tweets should discuss the author’s adoption of social distancing measures due to their infection, such as self-isolation or notifying close contacts.
11	Substantial evidence	Tweets should contain substantial evidence, such as medical records, official notices, or other documents validating the author’s COVID-19 infection.
12	Exclusion of transmission	Tweets emphasizing that the author did not transmit the virus to others may indicate a self-reported infection.
13	Consensus annotation	If 3 or more annotators provide inconsistent annotations for a particular tweet, a discussion among all annotators should be initiated to reach a final consensus.

^a^PCR: polymerase chain reaction.

### Ethical Considerations

We have prioritized privacy and ethical considerations in our use of self-reported COVID-19–related tweets from Twitter, which, while publicly available, are processed with strict anonymization measures to ensure that no personally identifiable information can be traced back to individuals. The ethical nuances of using such data are addressed by considering users’ implied consent when posting self-reports on a public platform. To safeguard data security throughout collection, annotation, and analysis, we have implemented encryption, controlled access, and other technical measures, ensuring that the data are strictly used for research purposes and never for commercial activities. Furthermore, recognizing the inherent noise and biases in social media data, we explicitly acknowledge the limitations of our dataset and analysis. To mitigate potential risks of misinterpretation or misuse, we emphasize that our findings are intended for population-level trend analysis rather than individual diagnosis, thereby maintaining the integrity and appropriate application of the results. This study is exempt from ethical approval because it only involves the analysis of aggregate data, ensuring that no individual privacy information is disclosed. All data have been fully deidentified, and appropriate anonymization measures have been applied to guarantee that no subject can be traced.

### Self-Reported COVID-19–Positive Model

To select text-classification models to determine whether a tweet is a self-reported COVID-19– positive tweet, we trained both traditional machine learning methods and fine-tuned LLMs. We divided the manually labeled dataset in which 80% (92,171/115,214) was used as the training set, 10% (11,521/115,214) as the validation set, and the remaining 10% (11,521/115,214) as the test set. We used word frequency, term frequency–inverse document frequency [54] vectors, and feature hashing vectors as methods for text feature extraction, and we adopted 10-fold cross-validation to ensure the reliability of the results.

For conventional machine learning models, we used both linear and nonlinear models to compare different classification approaches. Because the amount of the manually annotated data were relatively small and the text data features were relatively simple, we experimented with machine learning methods such as naive Bayes [55], SVMs [56], and LR [57]. In the SVM method, we used the radial basis function as the kernel function and set the penalty parameter to 1.2. We used an L2 regularization as the penalty term in LR, with a regularization strength parameter (C) set to 1.0. As for LLMs, we used BERT [49], robustly optimized BERT pretraining approach (RoBERTa) [58], extreme language model [59], GPT-2 [60], Bigscience Large Open-science Open-access Multilingual language model (BLOOM) [61], and Llama-2 [62] for training. These LLMs are pretrained on large datasets using the masked language model objective for BERT and RoBERTa; the permuted language model objective for extreme language model; and the causal language modeling objective for GPT-2, BLOOM, and Llama-2. Because GPT-2, BLOOM, and Llama-2 are generative models, they may generate nonuniform results, rendering result interpretation difficult. These pretrained LLMs were originally designed to process general text of varying lengths. Through fine-tuning these LLMs on tweets, which are inherently short texts, we adapted them to better handle short text-classification tasks. We selected models pretrained on English corpora to ensure appropriate language understanding. We borrowed the idea from previous work [63], leveraging latent representations from LLMs for supervised label prediction. Hence, we designed different LLM-based classifiers integrated with various LLMs and fully connected neural networks. Precisely, each LLM serves as a backbone for encoding the tweets instead of generating text. A fully connected neural network was integrated on top of each LLM for stably accurate detection of self-reported COVID-19 cases. Unlike traditional machine learning methods requiring feature preprocessing, the LLM-based classifiers take only the text of a tweet as input. The specific parameters of the machine learning methods and LLMs can be found in Multimedia Appendices 6 and 7.

To prevent catastrophic forgetting and ensure that LLMs have a broad understanding of self-reported COVID-19 cases during the training stage, we used low-rank adaptation (LoRA) [64] to fine-tune the LLM-based classifiers. LoRA enables the parameters of a model to learn effectively by introducing trainable rank decomposition matrices into the transformer architectures in the LLMs. To achieve this reparameterization, we modified the projection matrices of query, key, value, and feedforward network modules within the self-attention mechanism of the transformer.

The LLM-based classifiers were trained end-to-end with the AdamW [65] optimizer with a cross-entropy objective function. During the training stage, the parameters introduced by LoRA within the pretrained model were updated with gradients, and all remaining parameters were frozen. Early stopping to monitor the accuracy of the validation dataset was implemented during training. All runs were trained on the Nvidia A100 graphical processing unit with a batch size of 5 for Llama-2 and 32 for other models.

We evaluated the performance of each model and chose the one that achieved the best results for predicting the daily number of self-reported confirmed cases. Subsequently, we applied a named entity recognition [66,67] method such as SpaCy [68] and BERT-based models to extract essential symptom-related keywords from the tweets. The model was trained or fine-tuned using a labeled dataset specifically curated for health-related text, which included annotations for COVID-19 symptoms such as fever, cough, fatigue, and loss of smell or taste. For the definition of symptoms, we referred to the descriptions of symptoms within the systematized nomenclature of medicine clinical terms [69] as shown in Table 2. To make our system operational, we deployed the trained model on a server. We used a script program to continuously collect COVID-19–related tweets from Twitter. The collected tweets were then analyzed using the deployed model, and the results were displayed weekly on the Covlab website. This provided users with up-to-date, analyzed information regarding COVID-19 development.

**Table 2:** Symptoms and their expression found in self-reported tweets.

Symptoms	Descriptions (with their IDs in clinical terms)
Fever	Febrile (386661006), fever (386661006), feverish (103001002), mill fever (85761009), hyperthermia (1197782006), hay fever (21719001), degrees Fahrenheit (258712004), temperature (722490005), high temperature (285717004), high body temperature (50177009), body temperature above reference range (50177009), increased body temperature (50177009), elevated temperature (50177009), raised temperature (50177009), increased skin temperature (17038008), feeling hot (373932008), feeling hot and cold (103002009), and feeling hot and sweaty (373939004)
Chills	Chills (43724002), chills and fever (274640006), chillness (43724002), shivering (43724002), shivering or rigors (248456009), rigor (38880002), brass founders’ ague (74800004), algor (425681008), shakes (26079004), shaking (26079004), trembling (267079009), cold (82272006), head cold (82272006), freeze (48103003), freezing (48103003), and frigid (48103003)
Sweating	Sweat (74616000), sweating (415691001), cold sweat (83547004), hot sweat (224962007), hemopoiesis (445961003), hidrosis (415691001), diaphoresis (52613005), perspiration (415691001), perspire (74616000), perspire profusely (74616000), started to perspire (74616000), perspire all over (74616000), perspire during sleep (74616000), excessive sweating (52613005), profuse sweating (52613005), and sweating profusely (52613005)
Runny nose	Sniffle (275280004), nose running (267101005), running nose (267101005), nose dripping (267101005), nasal discharge (267101005), and snotty (267101005)
Nasal congestion	Nasal congestion (68235000), congested nose (68235000), stuffed-up nose (68235000), congestion (85804007), stuffed-up nose (68235000), stuffed nose (68235000), rhinobyon (68235000), nasal obstruction (232209000), nasal airway obstruction (232209000), and stuffiness (232209000)
Nosebleed	Nosebleed (249366005), nose bleeds (249366005), nose bleeding (249366005), bleeding from nose (249366005), nosebleed (249366005), nasal hemorrhage (249366005), epistaxis (249366005), and nasal hemorrhage (249366005)
Cough	Cough (49727002), coughing (49727002), nonproductive cough (11833005), hacking cough (59994004), tussiculation, dry cough (11833005), persistent cough (284523002) acute cough (49727002), bad cough (49727002), coughing all night (161933007), evening cough (161933007), morning cough (161932002), coughing up blood (66857006), coughing and deep breathing (371605008), and begma (49727002)
Headache	Headache (25064002), migraine (37796009), sick headache (193028008), tension-type headache (398057008), and cluster-headache syndrome (193031009)
Sneezing	Sneeze (76067001), sneezing (76067001), sneezing symptom (162367006), sternutation (76067001), niesen (76067001), and achoo (76067001)
Eye pain	Eye pain (41652007), pain in eye (41652007), ocular pain (41652007), ocular headache (86925001), ocular dryness (162290004), dry eye (162290004), cephalalgia (25064002), diplopia (24982008), double vision (24982008), and eyelid edema (89091004)
Loss of taste or smell	Smell (397686008), taste (397627001), lost sense of smell (44169009), absent smell (44169009), sense of smell absent (44169009), anosmia (44169009), can’t smell (44169009), smell nothing (44169009), disorder of taste (399993004), loss of taste (36955009), absence of sense of taste (36955009), and ageusia (36955009)
Sputum	Sputum (45710003), expectoration (45710003), productive cough (28743005), productive cough–green sputum (161924005), productive cough–yellow sputum (161925006), and phlegm (52024008)
Shortness of breath	Respiratory disorder (50043002), respiratory disease (50043002), breath (11891009), shortness of breath (267036007), dyspnea (267036007), short breath (267036007), breathless (267036007), difficulty in breathing (230145002), breathing difficult (230145002), labored breathing (248549001), difficulty breath (230145002), and breathing painful (75483001)
Sore throat	Sore throat (267102003), throat sore (162388002), throat pain (162388002), pain in throat (162397003), and pain throat (162397003)
Dizziness	Dizzy (267102003), throat soreness (267102003), dizzy spells (315018008), and dizziness (404640003)
Intolerance to light	Intolerance of light (1285284009), photophobia (409668002), eye sensitive to light (1285284009), light sensitive (1285284009), and light sensitivity (1285284009)
Hearing findings	Pains of ears (301354004), earache (301354004), otalgia (301354004), ears pop (162346006), popping sensation in ear (162346006), tinnitus (60862001), and noise in ears (60862001)
Loss of appetite	Poor appetite (64379006), decrease in appetite (64379006), inappetence (64379006), lost my appetite (64379006), loss of appetite (79890006), no appetite (79890006), anorexia (79890006), and off food (79890006)
Hallucinations	Hallucinations (7,011,001), illusion (5,152,006), illusionary (5,152,006), auditory hallucination (45150006), visual hallucination (64269007), and see things (64269007)

### Phase Difference Calculation

Because it took time to provide the official report, the COVID-19 occurrence derived from Twitter (ie, predicted curve) was expected ahead of the official report dates (ie, actual curve). Our research used the Hilbert transform (HT) [70] method to calculate the phase difference between the actual and predicted curves. HT is a method used for analyzing time-varying signals [71]. It can transform a real-valued signal into a complex-valued signal, rendering it convenient for phase analysis. In signal analysis, HT is often used to calculate the instantaneous phase of a signal, which can be used to compare the phase difference between 2 signals. HT was selected for its ability to compute instantaneous phase and amplitude in the time domain, making it ideal for analyzing temporal alignment and detecting phase shifts between predicted and actual signals. Unlike Fourier transform, which is limited by global frequency components, HT offers a more accurate and intuitive method for capturing time-varying phase relationships. It is particularly useful for nonstationary signals, as it can analyze phase dynamics without assumptions of stationarity or periodicity. The phase spectrum of Fourier transform may have discontinuous jumps in some cases, which can lead to incorrect results when calculating phase differences. HT can accurately calculate the instantaneous phase of a signal and avoid this problem.

The daily predicted cases curve *f*(t) and the daily actual cases curve *g*(t) share the same time sequence. To calculate the phase difference between them by performing the HT, we can first transform them into their complex-valued signals:



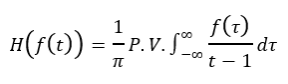



**(1)**



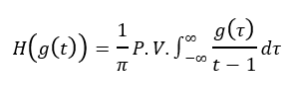



**(2)**

Here, [H] represents the HT operator, and *i* is the imaginary unit. We can then calculate the instantaneous phase of each signal, usually using the *arctan* function to compute the phase angle:



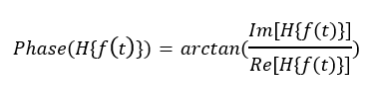



**(3)**



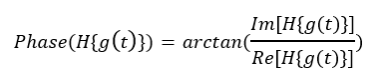



**(4)**

Finally, we subtracted the phase angles of the 2 signals to obtain the phase difference :



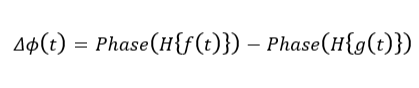



**(5)**

In addition to calculating the phase difference between the 2 curves, we also conducted stationarity tests on both curves. Stationarity verification is an important step in time-series analysis and is used to determine whether a time series is stationary. We used 3 methods, the augmented Dickey-Fuller test [72], the Kwiatkowski-Phillips-Schmidt-Shin test [73], and the Phillips-Perron test [74], to verify the stationarity of the 2 curves. We used the TimesNet [75] approach from prior research to predict the current trends in COVID-19 development based on the time-series relationships between the self-reported case numbers and the actual case numbers.

### Evaluation of the Model

Our evaluation of the model used the following methodology. True positive is the number of correct predictions in positive samples, false positive is the number of incorrect predictions in positive samples, true negative is the number of correct predictions in negative samples, and false negative is the number of incorrect predictions in negative samples. Precision is the proportion of positive predictions in all positive samples. Precision is defined as follows:



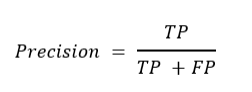



**(6)**

Recall is the proportion of correct predictions in the total samples. Recall is defined as follows:



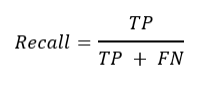



**(7)**

Accuracy is defined as the percentage of correctly predicted results out of the total sample.



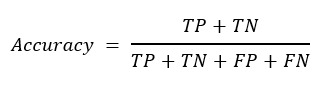



**(8)**

*F*_1_-score is defined as follows:



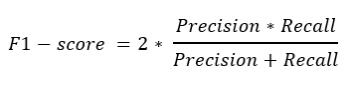



**(9)**

To measure the performance under the unbalanced data distribution, in this work, we used the precision-recall (PR) curve and ROC curve to display the performance. The ROC curve is a curve of sensitivity versus 1–specificity on all possible prediction thresholds. Similarly, the PR curve plots precision versus recall on all possible prediction thresholds. In addition, average precision (AP) and area under the curve (AUC) derived from the PR curve and the ROC curve are also generated for quantitative comparisons in this work.

## Results

### Model Performance for Self-Reporting COVID-19 Cases

**Figure 2:**
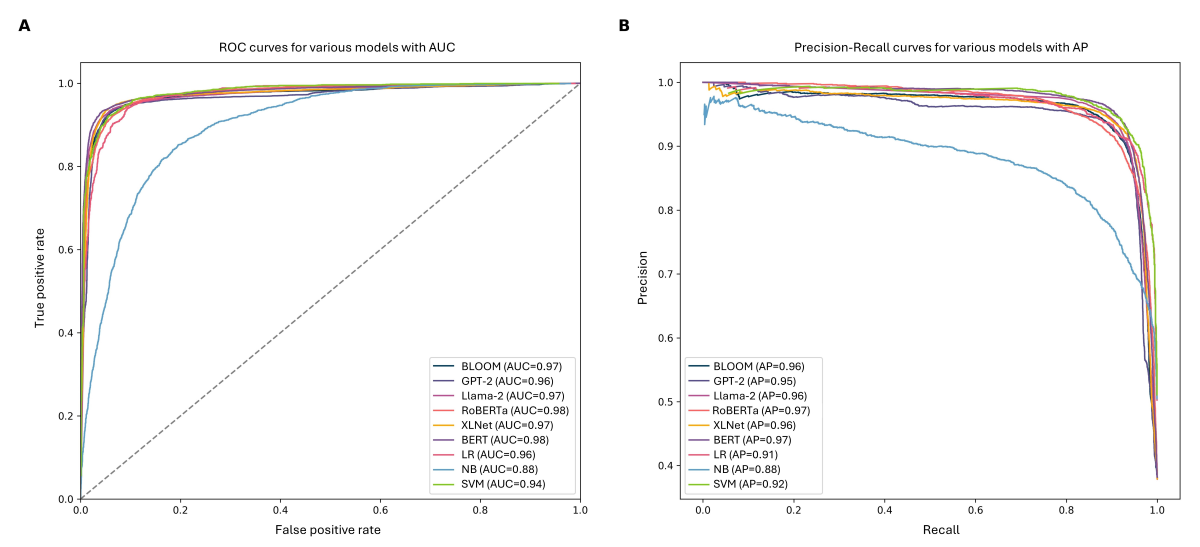
Performance of various machine learning methods. (A) Receiver operating characteristics (ROC) curve with area values. The area under the curve (AUC) values, provided in the legend, quantify the overall performance of the models, with higher values indicating superior discriminative ability. (B) Precision-recall curve with average precision. AP: average precision; BERT: Bidirectional Encoder Representations from Transformers; BLOOM: Bigscience Large Open-science Open-access Multilingual language model; Llama-2: large language model meta AI 2; LR: logistic regression; NB: naive Bayes; RoBERTa: robustly optimized BERT pretraining approach; SVM: support vector machine; XLNet: extreme language model.

We evaluated the models with AUC, AUC-PR, accuracy, precision, recall, and *F*_1_-score. RoBERTa and BERT achieved the best performance with an AUC of 0.98 and an AP of 0.97, as depicted in Figure 2. Notably, all LLMs outperformed traditional machine learning models in AUC and AP, exhibiting an AUC gain from 0.01 to 0.10 and an AP gain from 0.07 to 0.09. According to the benchmark results in Table 3, BLOOM performed the best compared with other models in accuracy and precision with 0.948 and 0.941, whereas the SVM outperformed others in recall and *F*_1_-score with 0.9362 and 0.9329, respectively. Combining AUC, accuracy, and recall, we believe that the BLOOM model has the best performance. Subsequently, we used the trained model to assist us in selecting self-reported positive tweets from all COVID-19–related tweets.

**Table 3:** Performance comparison of various machine learning models and large language models.

			Accuracy (%)		Precision (%)		Recall (%)		*F*_1_-score (%)
**Machine learning**									
	Naive Bayes [55]	86.86		65.62		52.43		58.29	
	Logistic regression [57]	92.57		91.73		92.64		92.18	
	Support vector machine [56]	93.62		92.96		*93.62*		*93.29*	
**Large language models**									
	BERT^a^ [49]	93.80		92.50		91.00		91.70	
	RoBERTa^b^ [58]	93.60		91.20		91.80		91.50	
	XLNet^c^ [59]	93.00		90.40		91.30		90.80	
	GPT-2 [60]	94.30		92.00		91.00		92.60	
	BLOOM^d^ [61]	*94.80*		*94.10*		92.00		93.00	
	Llama-2^e^ (7b) [62]	94.20		93.30		91.30		92.30	

^a^BERT: Bidirectional Encoder Representations from Transformers.

^b^RoBERTa: robustly optimized BERT pretraining approach.

^c^XLNet: extreme language model.

^d^BLOOM: Bigscience Large Open-science Open-access Multilingual language model.

^e^Llama-2: large language model meta AI 2.

### Predicted Trend of COVID-19 Cases

From January 1, 2020, to April 1, 2023, we used the trained BLOOM model to evaluate all the collected COVID-19–related tweets and filtered out the tweets that the model predicted as self-reported positive cases of COVID-19. Among 7.3 million COVID-19–related tweets, we identified 317,500 self-reported tweets. Using unique user IDs, we considered multiple tweets reporting a COVID-19 diagnosis by the same user to be a single case, resulting in 262,278 unique self-reported cases. The IDs of these unique confirmed users have been stored separately in an in-house database named the COVID-19 patient database (CPD), and the daily predicted number of confirmed cases by the model has been stored according to Coordinated Universal Time.

**Figure 3:**
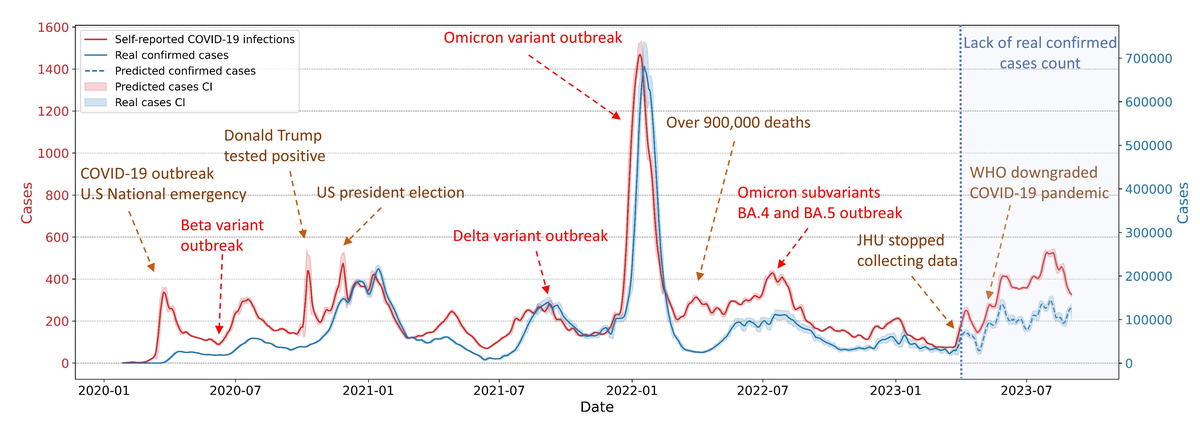
Real cases and predicted cases curves. The blue curve represents the actual daily confirmed cases, and the red curve represents the daily predicted cases. The shaded areas above and below the red and blue curves represent the CI. The red text represents key events during the outbreak, and the brown text represents the time the variant appeared. The blue shaded area on the right side represents the period during which actual confirmed case data are missing. The solid red line represents the daily self-reported COVID-19 infection numbers, and the blue dotted line represents the predicted actual infection numbers. JHU: Johns Hopkins University; WHO: World Health Organization.

To compare the predicted daily case counts with the actual daily case counts, we obtained the actual daily case counts from public platforms such as Johns Hopkins University and *The New York Times*. Then, we plotted the actual daily case count and predicted case count on a curve, as shown in Figure 3. The blue line represents the daily actual case counts, and the red line represents our predicted case counts. The red text in the figure describes key events during the outbreak, and the brown text represents the time the variant appeared. We used the HT method to calculate the phase difference between the two curves and found that the predicted curve was leading the actual curve by approximately 7.63 days. The Augmented Dickey-Fuller test results indicated values below the critical values of 1%, 5%, and 10%, accompanied by simultaneous *P* values of <.001 and <.001, which rejects the hypothesis of the existence of a unit root. In addition, both the Phillips-Perron and Kwiatkowski-Phillips-Schmidt-Shin tests exhibited *P* values <.05. Collectively, the outcomes from these 3 tests consistently pointed toward the smoothness of the time series under scrutiny as shown in Multimedia Appendix 8.

There are 2 distinct peaks in the red curve. We examined the data for the first peak of the predicted curve on October 2, 2020, and the second peak on November 21, 2020. We found that the first peak on October 2, 2020, was due to then US President Donald Trump’s tweet announcing his positive COVID-19 test result, which triggered many Twitter users to also report their self-diagnosed cases on Twitter. On that day, there were 1495 tweets related to self-reported positive cases. As for the second peak in the prediction curve, we examined the relevant tweets on that day and found that most of them were related to the US election results. Many users tweeted about their infection status and discussed the US epidemic-prevention policies. There were 973 tweets on that day regarding self-reported cases of COVID-19 infection out of all tweets.

### Symptoms

**Figure 4:**
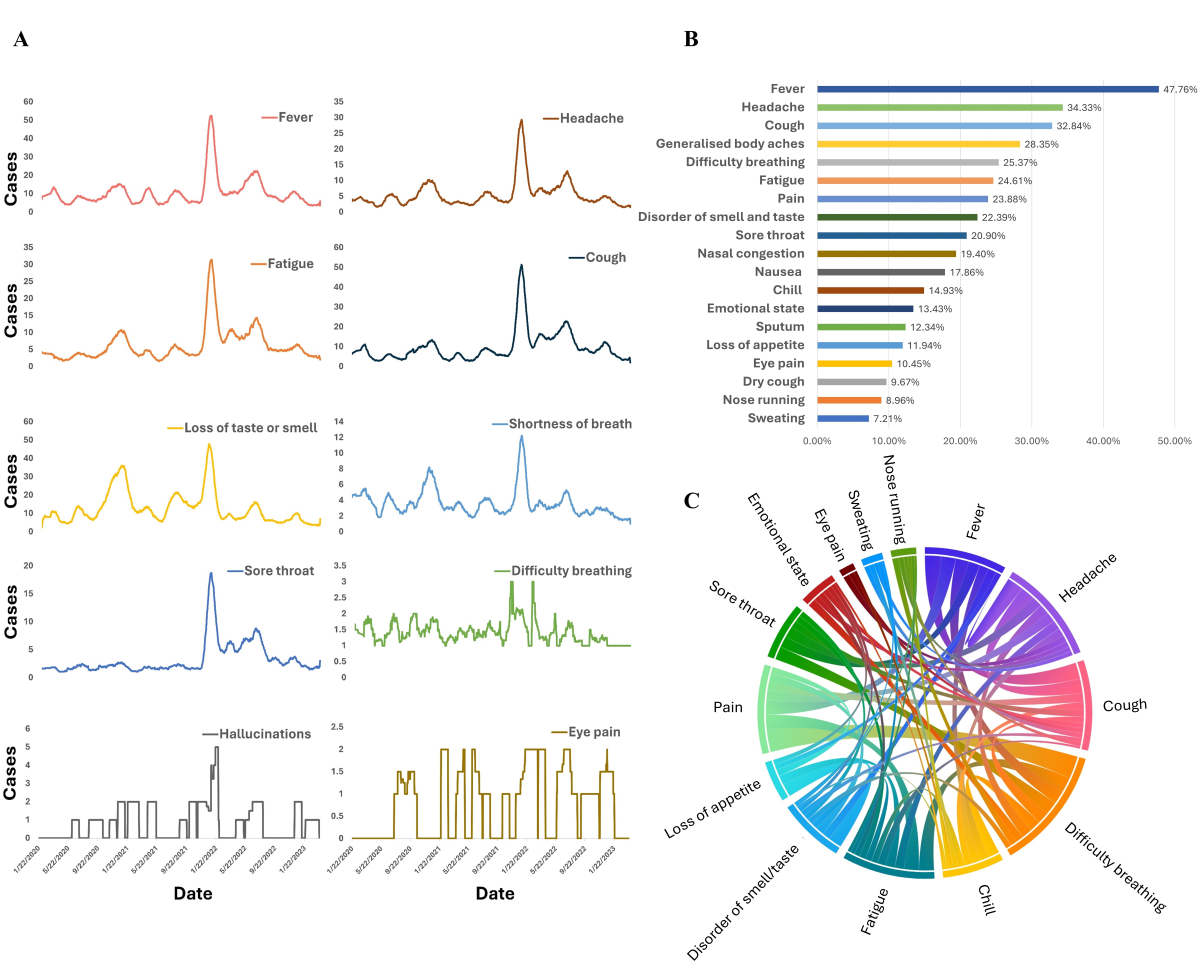
All symptoms mentioned by self-reporting tweets and the correlations among symptoms. (A) represents the number of mentions of COVID-19 symptoms in self-reported tweets over time. (B) represents the percentage of symptoms in all self-reporting COVID tweets, and multiple symptoms can be mentioned in 1 tweet. (C) represents the correlations among symptoms mentioned by the same user. The width of the line between 2 symptoms represents the number of tweets that mention both symptoms.

We extracted the historical tweets of users from the CPD using their unique user IDs. The temporal scope of these tweets spanned a period from 1 month preceding the self-reported date of symptom onset to 9 months after the diagnosis date, encompassing a total duration of 10 months. Within a cohort of 24,316 reporting COVID-19 symptoms, an analysis of historical tweets identified the top 10 symptoms. Figure 4A plots the temporal frequency of COVID-19 symptom mentions, providing insight into how symptom prevalence evolved over time. Notably, *fever*, *headache*, *fatigue*, and *cough* emerged as consistently common symptoms. The trends observed in these symptoms closely parallel the overall trend in confirmed COVID-19 cases. We also identified that the onset of symptoms such as *loss of taste or smell* and *shortness of breath* first became prominent in September 2020, possibly correlating with the emergence of the Beta variant. Similarly, the prevalence of *sore throat* spiked in late 2021, potentially aligning with the rise of the Omicron variant. The symptom *difficulty breathing* maintained a steady presence across the timeline. Notably, less commonly reported symptoms, such as *hallucinations* and *eye pain*, not currently recognized by the US Centers for Disease Control and Prevention (CDC), appeared sporadically in user reports, suggesting their rarity in COVID-19 cases. As shown in Figure 4B, these were *fever* (11,613/24,316, 47.76% mentioned), *headache* (8347/24,316, 34.33%), *cough* (7985/24,316, 32.84%), *generalized body* aches (6893/24,316, 28.35%), *difficulty breathing* (6169/24,316, 25.37%), *fatigue* (5984/24,316, 24.61%), *pain* (5806/24,316, 23.88%), *disorder of smell and taste* (5444/24,316, 22.39%), and *sore throat* (5082/24,316, 20.9%), listed in descending order. Notably, all symptoms except for *eye pain* (2541/24,316, 10.45%) aligned with those recognized by the CDC. Additional symptoms, such as *lethargy* (2176/24,316, 8.95%), *dizziness* (1451/24,316, 5.97%), and *hallucinations* (1086/24,316, 4.47%), although mentioned by a minority group, are not currently acknowledged as COVID-19 symptoms by the CDC.

In the dataset of historical tweets from diagnosed individuals, we observed instances in which a patient mentioned multiple symptoms concurrently. To quantify this, we calculated the frequency of cooccurrence of any 2 symptoms and constructed a dependency graph to illustrate these relationships. Figure 4C elucidates the correlations among various symptoms, highlighting that most individuals with the infection reported experiencing a constellation of related symptoms, such as headache, cough, and fever. Furthermore, Multimedia Appendix 9 presents a heat map that visualizes the Pearson correlation coefficients [76] among these symptoms, offering a quantitative view of their interdependencies. To visually represent the range of self-reported symptoms, we used a word cloud. This graphical representation provides an immediate overview of the symptomatology as expressed by the users in our dataset.

### Reinfection and Rehabilitation

**Figure 5:**
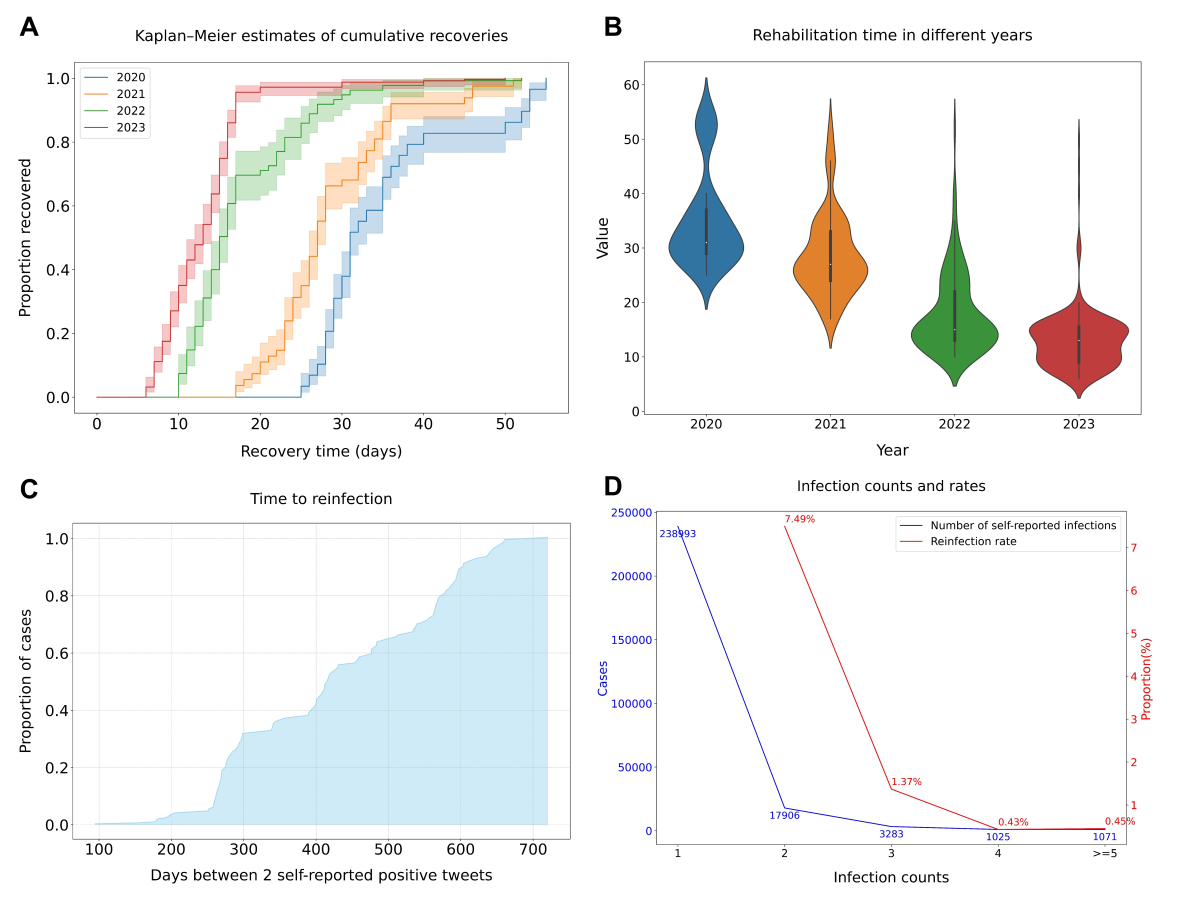
Overview of reinfections and recovery. (A) Kaplan-Meier estimates of cumulative recoveries. (B) Rehabilitation days in different years. (C) Time to reinfection for 238,993 individuals. (D) Reinfection cases and rates.

We analyzed historical tweets from users who self-reported COVID-19 infections, identifying 723 individuals who shared their recovery experiences. The annual breakdown of these individuals is as follows: 174 in 2020, 163 in 2021, 135 in 2022, and 251 in 2023. The duration of recovery was primarily inferred from the period mentioned in their tweets. In instances in which the recovery period was not explicitly stated, we computed it by calculating the interval between the date of confirmed diagnosis and the date of reported recovery. The data on self-reported recovery durations have been depicted through the Kaplan-Meier recovery curve [77], as illustrated in Figure 5A. This graphical representation reveals a gradual decrease in recovery time for patients with COVID-19 from 2020 to 2023. Specifically, in 2020, most patients reported a recovery period of around 30 days, with very few recovering in <30 days. In contrast, by 2023, the trend had shifted significantly, with most individuals reporting a recovery within approximately 12 days, despite a minority group still experiencing recovery periods extending beyond 30 days. Figure 5B presents a comprehensive overview of the evolution of recovery periods from 2020 to 2023. In addition, this figure suggests that the prevalent COVID-19 cases in 2023 were predominantly mild, indicating a possible decrease in the virulence of the virus over time.

In this study, we defined a recurrent COVID-19 infection in an individual as a self-reported reinfection occurring >30 days after the initial confirmed positive diagnosis. We meticulously tracked the historical tweets of all confirmed patients in CPD. Figure 5C presents the distribution of the intervals between the first and second infections alongside the corresponding case counts. This analysis reveals a relatively low likelihood of a repeat infection within 180 days. Most patients who had recovered from an initial infection reported a second infection approximately 260 days later. Moreover, repeat infections occurring between 300 and 600 days after recovery were also relatively frequent. The longest interval between repeat infections documented in our study extended to 720 days. Of 262,278 patients who self-reported a positive COVID-19 test result, 91.12% (n=238,993) indicated a single infection event, and 6.83% (n=17,906) reported 2 infections. A smaller subset, comprising 1.25% (n=3283) of individuals, reported 3 infections; 0.39% (n=1025) indicated 4 infections; 0.17% (n=445) reported 5 infections; 0.11% (n=301) of individuals reported 6 infections; and 0.12% (n=325) indicated ≥7 infections. Remarkably, the highest number of reported reinfections was 9, with 7 individuals documenting their ninth infection. Figure 5D shows that among the 238,993 patients with a single infection, 7.49% (17,906/238,993) reported a second infection. A further breakdown shows that 1.37% (3283/238,993) reported a third infection, 0.43% (1025/238,993) reported a fourth infection, and 0.45% (1071/238,993) reported experiencing ≥5 infections. We also performed a statistical analysis of the time intervals between infections among users with multiple infections.

### Distribution of Cases

**Figure 6:**
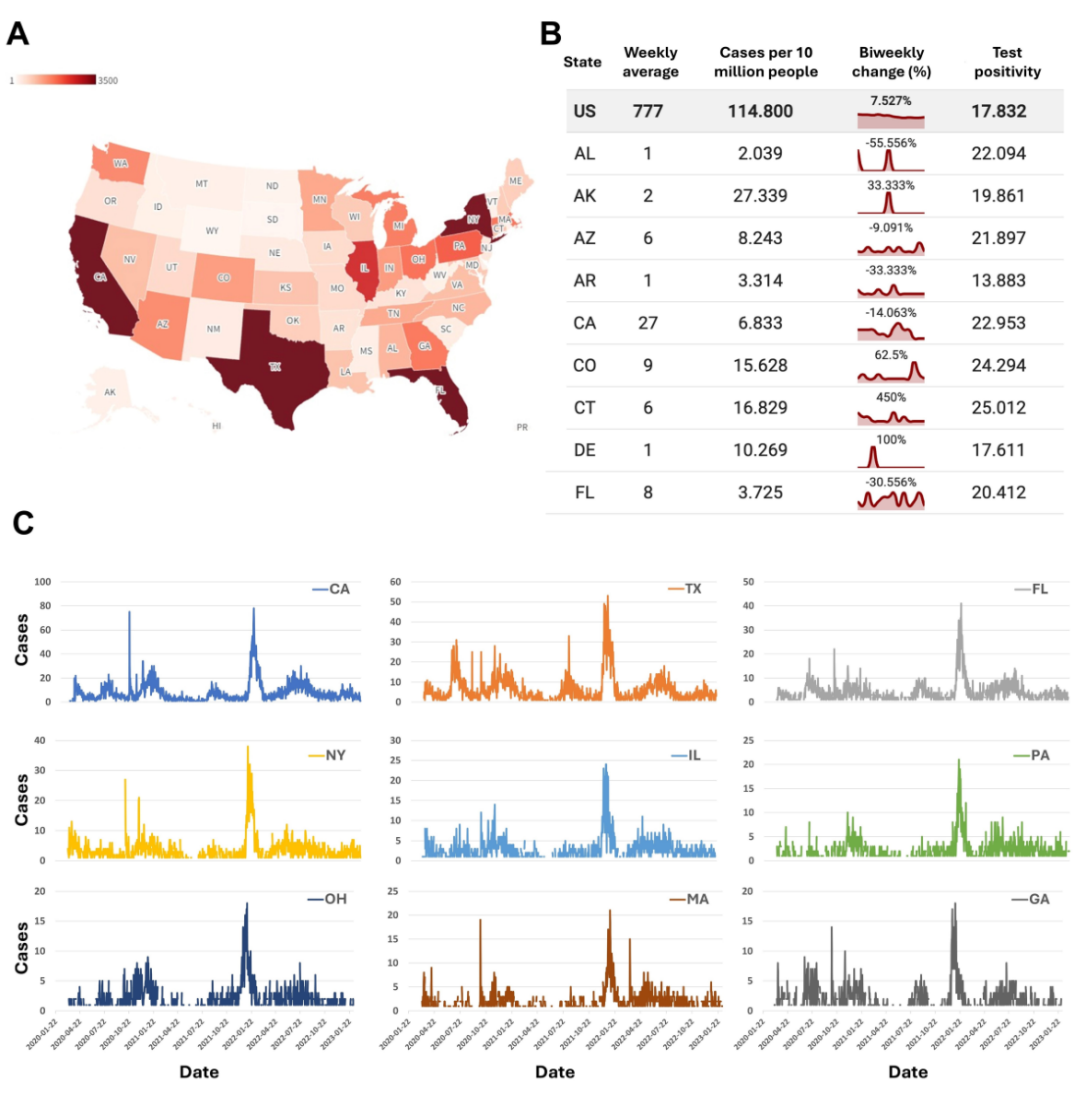
Cases across the United States and by state. (A) Self-reported number of infections in each state, with darker colors representing higher numbers. (B) Self-reported weekly number of infections, number of infections per 10 million people, change in trend over the previous 2 weeks, and positivity rate for a subset of states (alphabetical order). (C) Infection curve for the top 20 states with the highest infection numbers.

We extracted the geographic locations of the diagnosed users in CPD, and the distribution of all confirmed patients is shown in Figure 6A [52]. California had the highest number of self-reported COVID-19 cases, with 8762 cases, followed by Texas (6619 cases), Florida (4245 cases), New York (3566 cases), Illinois (2649 cases), Pennsylvania (2032 cases), Ohio (1868 cases), Massachusetts (1793 cases), Georgia (1785 cases), and Michigan (1677 cases), in descending order. Alabama and Northern Mariana Islands had the least data, with only 1 self-reported case in each state.

The details of confirmed cases in each state are shown in Figure 6B, in which we provide the average case counts for the entire country and each state as well as the number of self-reported cases per 10 million people per week, the trend over the past 2 weeks, and the positivity rate of each state. For example, we can see that California had the most self-reported COVID-19 cases in the past week, with at least 29 people reporting positive test results. Approximately 7.34 people per 10 million reported self-diagnosed COVID-19–positive status, and the average positivity rate of self-reported cases was 26.69%. However, compared with the previous 2 weeks, the number of self-reported COVID-19 cases decreased by 10.93%. Due to insufficient data, the trend of changes in the past 14 days was unavailable for several states, such as the Virgin Islands and Wyoming.

We also plotted the time-varying curves for the confirmed cases in the top 20 states in terms of confirmed cases, as shown in Figure 6C and Multimedia Appendix 10. It is evident that the changes in the number of confirmed cases in the top 4 states with the highest number of cases closely resemble the overall trend in the United States. Some states, such as Washington, Arizona, Washington DC, and Indiana, exhibited relatively consistent changes in the number of confirmed cases over time, whereas states such as Nevada, Colorado, Alabama, and Michigan had less-consistent curves, with some dates showing no reported cases. The variation in results could have been influenced by the differing numbers of Twitter users in each state.

### Covlab Visualization Website

To disseminate timely information to the public and policy makers, we have launched a visualization website, Covlab, which features our trained models and a comprehensive data pipeline. This platform consists of an automated script sequence designed to update the data weekly. The home page of Covlab offers users real-time access to the total number of tweets collected to date, including those self-reported as COVID-19–positive. On the Graphs page, users can explore the most recent weekly growth trends in COVID-19 cases alongside predictive models of actual confirmed cases. This section also enables monitoring of infection trends across various states in the United States as well as the prevailing symptom patterns observed to the current date. Furthermore, Covlab displays the proportions of reinfections and recovery periods, with these metrics also being refreshed weekly. The website’s dynamic graphs and tables serve as a valuable resource, providing the public with up-to-date information on the ongoing evolution of COVID-19, including insights into the symptomatic expressions of emerging variants.

## Discussion

### Principal Findings

The analysis of self-reported COVID-19 tweets offers a valuable perspective, reflecting the actual progression of the pandemic to a considerable degree. The voluminous data generated by self-reporting individuals on Twitter augment clinical datasets, offering a complementary avenue for long-term observation and tracking. This approach is particularly beneficial in bridging the data gap inherent in in-home self-testing scenarios. Our research indicates that reinfections are relatively common, with the likelihood of multiple infections diminishing over time. Moreover, this dataset has proven instrumental in identifying potential new symptoms that were not initially associated with COVID-19, such as lethargy and hallucinations, offering an early warning system for evolving clinical presentations. In addition, the approach has enabled the detection of emerging variants through geospatial clustering of self-reported cases exhibiting distinctive symptom combinations. This tracking capability enhances preparedness and enables timely public health interventions. The breakdown by state of self-reported COVID-19 tweets facilitates more efficient tracking of disease trends. Furthermore, the approach used in this study could potentially serve as a general pipeline for researching and analyzing other infectious diseases.

### Symptoms and Sequelae Study

Within the subset of Twitter users who self-reported as COVID-19 positive, numerous accounts contained detailed descriptions of symptoms experienced during the infection. These firsthand accounts are a rich source of information. We extracted and analyzed the symptomatology mentioned in these tweets, compiling a list of the most prevalent symptoms reported by patients. Our intriguing findings closely mirror the commonly acknowledged COVID-19 symptoms listed by the CDC. However, some symptoms mentioned by a subset of patients in their tweets are not yet recognized as typical by the CDC. This discrepancy highlights the significant role of our study in identifying potential new symptoms of COVID-19 that have not been widely recognized. Such findings could provide valuable guidance for further clinical investigation into these symptoms and their association with different COVID-19 variants. In addition, the CPD established through this study offers a robust framework for long-term patient tracking. This database not only complements existing clinical data but also provides an invaluable resource for the study of post–COVID-19 sequelae and post–COVID-19 condition symptoms, thereby enhancing our understanding of the virus’s long-term impacts.

### Comparison With Traditional Tracking Tools

Traditional clinical data–driven tools, while offering high specificity by relying on confirmed diagnoses, often underrepresent asymptomatic or mild cases. In contrast, our approach leverages self-reported data from social media platforms, such as Twitter, to capture a broader spectrum of cases, including those not reported in clinical settings. Although this may introduce noise, advanced filtering and NLP techniques effectively mitigate inaccuracies, ensuring meaningful insights. Furthermore, clinical data collection and reporting are often delayed due to testing and diagnosis, whereas our tool enables near–real-time monitoring by analyzing self-reported cases as they are posted, facilitating rapid identification of trends and potential hot spots. In terms of coverage, traditional clinical tracking tools may lack granularity, particularly in regions with limited health care infrastructure or reporting capabilities. Using social media, our tool significantly enhances coverage, incorporating underrepresented populations and geographies to provide a more comprehensive view of the pandemic’s spread. A key contribution of this approach is the integration of self-reported data, which captures personal narratives, symptom progression, and public sentiment, thereby complementing clinical data to offer a richer and more dynamic understanding of the pandemic. Importantly, while our tool excels in timeliness and coverage, its true potential lies in synergy with clinical data–driven tools. We propose future efforts to integrate these approaches, enabling cross-validation and improving overall accuracy.

### Limitations and Future Work

Although our method and website are highly useful, there are some limitations. First, Twitter has a limited quota of public APIs, which renders the platform hugely expensive to run. Second, potential biases in data collection and the varying distribution of Twitter users across different states may impact the predictive accuracy. Furthermore, external factors, particularly notable events in the United States that occurred in 2020, influenced our prediction outcomes. For instance, the 2 notable peaks in self-reported cases observed in 2020 did not necessarily correlate with an actual increase in infections. Instead, these peaks were primarily driven by external events, prompting a surge in infection reporting on Twitter on those specific days. Another significant constraint is the veracity of the information in tweets. However, despite their questionable reliability, these data offer informative trend analyses and hypotheses valuable for future research and validation. Our framework demonstrates significant versatility, with potential applications extending beyond COVID-19 to monitor other self-reported illnesses, such as influenza and respiratory syncytial virus, as well as chronic conditions such as diabetes and mental health issues. Its modular design facilitates easy adaptation by allowing the customization of keywords and analytical strategies tailored to specific diseases. To enhance its functionality, we propose integrating Twitter data with other real-time health data sources, such as wearable devices and electronic health records, enabling a more comprehensive approach to health trend monitoring. In this integration, social media signals would serve as an initial screening mechanism, effectively complementing clinical data. In future work, we plan to integrate self-reported data from other social platforms, such as Reddit, to reduce data limitations and biases, thereby broadening the scope of our analysis. This expansion will support the development of a platform capable of predicting COVID-19 trends in real time based on self-reported content. Furthermore, we aim to apply this pipeline to other infectious diseases that may emerge in the future, facilitating the understanding and tracking of their development and trends. To maintain robustness and accuracy, we plan periodic retraining of the model using updated data and incorporating LLMs to better capture nuanced expressions in self-reported content. A user feedback mechanism will also be implemented to address false positives and negatives. In addition, we aim to expand the tool’s global applicability by automating data pipelines to support content in multiple languages, with a particular focus on underrepresented regions. This global expansion will enable the tool to capture disease trends across diverse geographic and cultural contexts, offering a more holistic view of global health dynamics.

### Conclusions

This study demonstrates the significant potential of using self-reported COVID-19 tweets from social media platforms for public health monitoring. By leveraging machine learning and NLP techniques, we developed a tool capable of identifying infection and recovery trends, providing valuable insights into disease spread and public behavior. Our findings contribute to the growing field of digital epidemiology, emphasizing that social media can serve as an effective complementary data source to traditional public health surveillance systems. Beyond COVID-19, this approach holds promise for monitoring other infectious diseases, mental health conditions, and chronic illnesses by adapting the model to new health-related keywords and contexts. Integrating such tools with existing health infrastructure could enhance early detection, improve situational awareness, and enable more proactive public health responses. However, the ethical considerations of using publicly available data and addressing biases inherent to social media platforms must be prioritized to ensure responsible use. Overall, this work highlights the evolving role of digital tools in public health informatics and presents opportunities for future research to further refine these methods. The integration of social media–based monitoring with traditional data systems could transform public health strategies, making them more adaptive, responsive, and inclusive of diverse population segments.

## Appendix

Multimedia Appendix 1 A list of keywords and hashtags used in data collection.

Multimedia Appendix 2 Stop word list.

Multimedia Appendix 3 Interannotator agreement metrics.

Multimedia Appendix 4 Data preprocessing process and annotation system.

Multimedia Appendix 5 An example of the target cohort.

Multimedia Appendix 6 Number of parameters for conventional machine learning and large language models.

Multimedia Appendix 7 Configuration for conventional machine learning and large language models.

Multimedia Appendix 8 Multiple stationarity verification methods and their results.

Multimedia Appendix 9 Heat map of Pearson correlation coefficients among symptoms and symptoms word cloud.

Multimedia Appendix 10 Infection curve for the top 20 states with the highest infection numbers.

## Abbreviations

AP: average precision
API: application programming interface
AUC: area under the curve
BERT: Bidirectional Encoder Representations from Transformers
BLOOM: Bigscience Large Open-science Open-access Multilingual language model
CDC: Centers for Disease Control and Prevention
CPD: COVID-19 patient database
HT: Hilbert transform
Llama-2: large language model meta AI 2
LLM: large language model
LoRA: low-rank adaptation
NLP: natural language processing
PCR: polymerase chain reaction
PR: precision-recall
RoBERTa: robustly optimized Bidirectional Encoder Representations from Transformers pretraining approach
ROC: receiver operating characteristic

## Data Availability

The aggregated datasets analyzed in this study are available from the corresponding author upon request. Manual labeling data can be viewed through the annotation website. Guest users can use the username *guest* and password *guest* to log into the system for data access. Actual and predicted daily cases are publicly available on the Covlab website [52].
