# Supplementary material for: Leveraging Large Language Models for Infectious Disease Surveillance—Using a Web Service for Monitoring COVID-19 Patterns From Self-Reporting Tweets: Content Analysis
Source: J Med Internet Res. 2025 Feb 20;27:e63190. doi: 10.2196/63190 (PMC11888100; doi:10.2196/63190)
Supplement: Multimedia Appendix 1 [file jmir_v27i1e63190_app1.docx]

**Table S1. A list of keywords and hashtags used in data collection**

| **Keywords** | **Hashtags** |
| --- | --- |
| I.* tested[ed] positive for [covid \| coronavirus \| covid19 \| covid-19] | #COVID |
| My.* [covid \| coronavirus \| covid19 \| covid-19].* symptoms | # Coronavirus |
| I covid diagnosis | #CoronaVirusUpdate |
| I got/infected covid | #COVID19USA |
| My covid result positive | #StayHomeStaySafe |
| I covid quarantine | #LongCovid |
| I covid confirmed | #CovidTesting |
