# Supplementary material for: Leveraging Large Language Models for Infectious Disease Surveillance—Using a Web Service for Monitoring COVID-19 Patterns From Self-Reporting Tweets: Content Analysis
Source: J Med Internet Res. 2025 Feb 20;27:e63190. doi: 10.2196/63190 (PMC11888100; doi:10.2196/63190)
Supplement: Multimedia Appendix 2 [file jmir_v27i1e63190_app2.docx]

**Table S2. Stop word list**

| Stop words |
| --- |
| “d”, “m”, “re”, “s”, “t, “ve”, “ZT”, “ZZ”, “a”, “as”, “able”, “about”, “above”, “abst”, “accordance”, “according”, “accordingly”, “across”, “act”, “actually”, “added”, “adj”, “adopted”, “affected”, “affecting”, “affects”, “after”, “afterwards”, “against”, “ah”, “ ain’t”, “all”, “allow”, “allows”, “almost”, “alone”, “along”, “already”, “also”, “although”, “always”, “am”, “among”, “amongst”, “an”, “and”, “announce”, “another”, “any”, “anybody”, “anyhow”, “anymore”, “anyone”, “anything”, “anyway”, “anyways”, “anywhere”, “apart”, “apparently”, “appear”, “appreciate”, “appropriate”, “approximately”, “are”, “area”, “areas”, “arise”, “around”, “as”, “aside”, “ask”, “asked”, “asking”, “asks”, “associated”, “at”, “auth”, “available”, “away”, “awfully”, “b”, “backed”, “backing”, “backs”, “be”, “became”, “because”, “become”, “becomes”, “becoming”, “been”, “before”, “beforehand”, “began”, “begin”, “beginning”, “beginnings”, “begins”, “behind”, “being”, “beings”, “believe”, “below”, “beside”, “besides”, “best”, “between”, “beyond”, “big”, “biol”, “both”, “brief”, “briefly”, “but”, “by”, “c”, “ca”, “came”, “case”, “cases”, “cause”, “causes”, “certain”, “certainly”, “changes”, “clear”, “clearly”, “co”, “com”, “come”, “comes”, “concerning”, “consequently”, “consider”, “considering”, “contain”, “containing”, “contains”, “corresponding”, “course”, “currently”, “d”, “date”, “definitely”, “describe”, “described”, “despite”, “differ”, “different”, “differently”, “discuss”, “do”, “does”, “doing”, “done”, “down”, “downed”, “downing”, “downs”, “downwards”, “due”, “during”, “e”, “early”, “ed”, “edu”, “effect”, “eg”, “eight”, “eighty”, “either”, “else”, “elsewhere”, “end”, “ended”, “ending”, “ends”, “enough”, “entirely”, “especially”, “et”, “et-al”, “etc”, “even”, “evenly”, “ever”, “everything”, “everywhere”, “ex”, “exactly”, “example”, “except”, “f”, “face”, “faces”, “fact”, “facts”, “far”, “felt”, “few”, “ff”, “fifth”, “find”, “finds”, “first”, “five”, “fix”, “followed”, “following”, “follows”, “former”, “formerly”, “forth”, “found”, “four”, “from”, “full”, “fully”, “further”, “furthered”, “furthering”, “furthermore”, “furthers”, “g”, “gave”, “general”, “generally”, “get”, “gets”, “getting”, “give”, “given”, “gives”, “giving”, “go”, “goes”, “going”, “gone”, “good”, “goods”, “got”, “gotten”, “great”, “greater”, “greatest”, “greetings”, “group”, “grouped”, “grouping”, “groups”, “h”, “had”, “happens”, “has”, “have”, “having”, “hed”, “hello”, “help”, “hence”, “here”, “here’s”, “hereafter”, “hereby”, “herein”, “heres”, “hereupon”, “hi”, “hid”, “high”, “higher”, “highest”, “hither”, “home”, “hopefully”, “how”, “howbeit”, “hundred”, “I’d”, “id”, “ie”, “ignored”, “immediate”, “immediately”, “importance”, “important”, “in”, “inasmuch”, “inc”, “indeed”, “index”, “indicate”, “indicated”, “indicates”, “information”, “inner”, “insofar”, “interest”, “interested”, “interesting”, “interests”, “into”, “invention”, “inward”, “is”, “it”, “it’ll”, “it’s”, “itd”, “its”, “itself”, “j”, “just”, “k”, “keep”, “keeps”, “kept”, “keys”, “kg”, “kind”, “km”, “knew”, “know”, “known”, “knows”, “l”, “large”, “largely”, “last”, “lately”, “later”, “latest”, “latter”, “latterly”, “least”, “less”, “lest”, “let”, “let’s”, “lets”, “like”, “liked”, “likely”, “line”, “little”, “long”, “longer”, “longest”, “look”, “looking”, “looks”, “ltd”, “m”, “made”, “mainly”, “make”, “makes”, “making”, “man”, “many”, “mean”, “means”, “meantime”, “meanwhile”, “member”, “members”, “men”, “merely”, “mg”, “might”, “million”, “miss”, “ml”, “more”, “moreover”, “most”, “mostly”, “mr”, “mrs”, “much”, “mug”, “must”, “n”, “nt”, “na”, “name”, “namely”, “nay”, “nd”, “near”, “nearly”, “necessarily”, “necessary”, “need”, “needed”, “needing”, “needs”, “neither”, “new”, “newer”, “newest”, “next”, “nine”, “ninety”, “no”, “nobody”, “non”, “none”, “nonetheless”, “noone”, “nor”, “normally”, “nos”, “not”, “noted”, “nothing”, “nowhere”, “number”, “numbers”, “o”, “obtain”, “obtained”, “obviously”, “of”, “off”, “often”, “oh”, “ok”, “okay”, “old”, “older”, “oldest”, “omitted”, “on”, “once”, “one”, “ones”, “only”, “onto”, “open”, “opened”, “opening”, “opens”, “or”, “ord”, “order”, “ordered”, “ordering”, “orders”, “other”, “others”, “otherwise”, “ought”, “out”, “outside”, “over”, “overall”, “owing”, “own”, “p”, “page”, “pages”, “part”, “parted”, “particular”, “particularly”, “parting”, “parts”, “past”, “per”, “perhaps”, “place”, “placed”, “places”, “please”, “plus”, “point”, “pointed”, “pointing”, “points”, “poorly”, “possible”, “possibly”, “potentially”, “pp”, “predominantly”, “presented”, “presenting”, “presents”, “presumably”, “primarily”, “problem”, “problems”, “promptly”, “proud”, “provides”, “put”, “puts”, “q”, “que”, “quickly”, “quite”, “qv”, “r”, “ran”, “rather”, “rd”, “readily”, “really”, “reasonably”, “recent”, “ref”, “refs”, “regarding”, “regardless”, “regards”, “related”, “relatively”, “research”, “respectively”, “resulted”, “resulting”, “results”, “right”, “room”, “rooms”, “run”, “s”, “said”, “same”, “saw”, “say”, “saying”, “says”, “sec”, “secondly”, “section”, “see”, “seeing”, “seem”, “seemed”, “seeming”, “seems”, “seen”, “sees”, “self”, “selves”, “sensible”, “sent”, “serious”, “seriously”, “seven”, “several”, “shall”, “shed”, “should”, “show”, “showed”, “showing”, “shown”, “showns”, “shows”, “side”, “sides”, “significant”, “significantly”, “similar”, “similarly”, “since”, “six”, “slightly”, “small”, “smaller”, “smallest”, “so”, “some”, “somebody”, “somehow”, “someone”, “somethan”, “something”, “sometime”, “sometimes”, “somewhat”, “somewhere”, “soon”, “sorry”, “specifically”, “specified”, “specify”, “specifying”, “state”, “states”, “still”, “stop”, “strongly”, “sub”, “substantially”, “successfully”, “such”, “sufficiently”, “suggest”, “sup”, “sure”, “t”, “take”, “taken”, “taking”, “tell”, “tends”, “th”, “than”, “thank”, “thanks”, “thanx”, “that”, “that’ll”, “that’s”, “that’ve”, “thats”, “the”, “themselves”, “then”, “thence”, “there”, there”ll, there”s, “there’ve”, “thereafter”, “thereby”, “thered”, “therefore”, “therein”, “thereof”, “therere”, “theres”, “thereto”, “thereupon”, “these”, “thing”, “things”, “think”, “thinks”, “third”, “this”, “thorough”, “thoroughly”, “those”, “thou”, “though”, “thoughh”, “thought”, “thoughts”, “thousand”, “three”, “throug”, “through”, “throughout”, “thru”, “thus”, “til”, “tip”, “to”, “took”, “toward”, “towards”, “tried”, “tries”, “truly”, “try”, “trying”, “ts”, “turn”, “turned”, “turning”, “turns”, “two”, “u”, “un”, “under”, “unless”, “unlike”, “unlikely”, “until”, “unto”, “up”, “upon”, “ups”, “us”, “use”, “used”, “useful”, “usefully”, “usefulness”, “uses”, “using”, “usually”, “uucp”, “v”, “value”, “various”, “very”, “via”, “viz”, “vol”, “vols”, “vs”, “w”, “want”, “wanted”, “wanting”, “wants”, “was”, “wasn’t”, “way”, “ways”, “we’d”, “wed”, “welcome”, “well”, “wells”, “went”, “were”, “what”, “what’ll”, “what’s”, “whatever”, “whats”, “when”, “whence”, “whenever”, “where”, “where’s”, “whereafter”, “whereas”, “whereby”, “wherein”, “wheres”, “whereupon”, “wherever”, “whether”, “which”, “while”, “whim”, “whither”, “who”, who”ll, who”s, “whod”, “whoever”, “whole”, “whom”, “whomever”, “whos”, “whose”, “why”, “widely”, “willing”, “wish”, “with”, “within”, “wonder”, “words”, “work”, “worked”, “working”, “works”, “world”, “would”, “wouldn’t”, “www”, “x”, “y”, “year”, “years”, “yes”, “yet”, “young”, “younger”, “youngest”, “z”, “zero”, “zt”, “zz” |
