# Supplementary material for: Leveraging Large Language Models for Infectious Disease Surveillance—Using a Web Service for Monitoring COVID-19 Patterns From Self-Reporting Tweets: Content Analysis
Source: J Med Internet Res. 2025 Feb 20;27:e63190. doi: 10.2196/63190 (PMC11888100; doi:10.2196/63190)
Supplement: Multimedia Appendix 3 [file jmir_v27i1e63190_app3.docx]

**Inter-annotator agreement metrics**:

We calculated the inter-annotator agreement using Fleiss’ kappa to obtain overall agreement among multiple annotators. The average kappa score was 0.936, indicating almost perfect agreement.

The formula for Fleiss' Kappa is:

$$\kappa=\frac{\bar{P}-\bar{P_{e}}}{1-\bar{P_{e}}}$$

Where:

- $\bar{P}$: Observed agreement across all samples:

$$\bar{P}=\frac{1}{N}\sum_{i=1}^{N} P_{i}$$

And $P_{i}$ is the observed agreement for sample $i$:

$$P_{i}=\frac{1}{n\left( n-1 \right)}\sum_{j=1}^{k} n_{ij}\left( n_{ij}-1 \right)$$

Where $n_{ij}$ is the number of annotators who assigned sample $i$ to category $j$, $n$ is the total number of annotators per sample, and $k$ is the number of categories.

- $\bar{P_{e}}$: Expected agreement if annotations were random:

$$\bar{P_{e}}=\sum_{j=1}^{k} p_{j}^{2}$$

Where $p_{j}$ is the proportion of all annotations assigned to category $j$:

$$p_{j}=\frac{1}{Nn}\sum_{i=1}^{N} n_{ij}$$
