# Supplementary material for: Leveraging Large Language Models for Infectious Disease Surveillance—Using a Web Service for Monitoring COVID-19 Patterns From Self-Reporting Tweets: Content Analysis
Source: J Med Internet Res. 2025 Feb 20;27:e63190. doi: 10.2196/63190 (PMC11888100; doi:10.2196/63190)
Supplement: Multimedia Appendix 4 [file jmir_v27i1e63190_app4.docx]

**Figure S1. Data preprocessing process and annotation system.** (A) the processing procedure of raw tweets. (B) the labeling system that highlights specific keywords, and allows annotators to filter content and leave remarks to any tweet. It can be accessed through<https://labelling.covlab.tech/>. The guest users can log in and access using the username "guest" and password "guest". In this annotation system, keywords such as 'I', 'COVID', and 'POSITIVE'. were highlighted in different colors to help annotators better understand the content of tweets. Annotators only need to click on the target tweets and submit the results in batches to complete the annotation process.
