# Supplementary material for: Leveraging Large Language Models for Infectious Disease Surveillance—Using a Web Service for Monitoring COVID-19 Patterns From Self-Reporting Tweets: Content Analysis
Source: J Med Internet Res. 2025 Feb 20;27:e63190. doi: 10.2196/63190 (PMC11888100; doi:10.2196/63190)
Supplement: Multimedia Appendix 5 [file jmir_v27i1e63190_app5.docx]

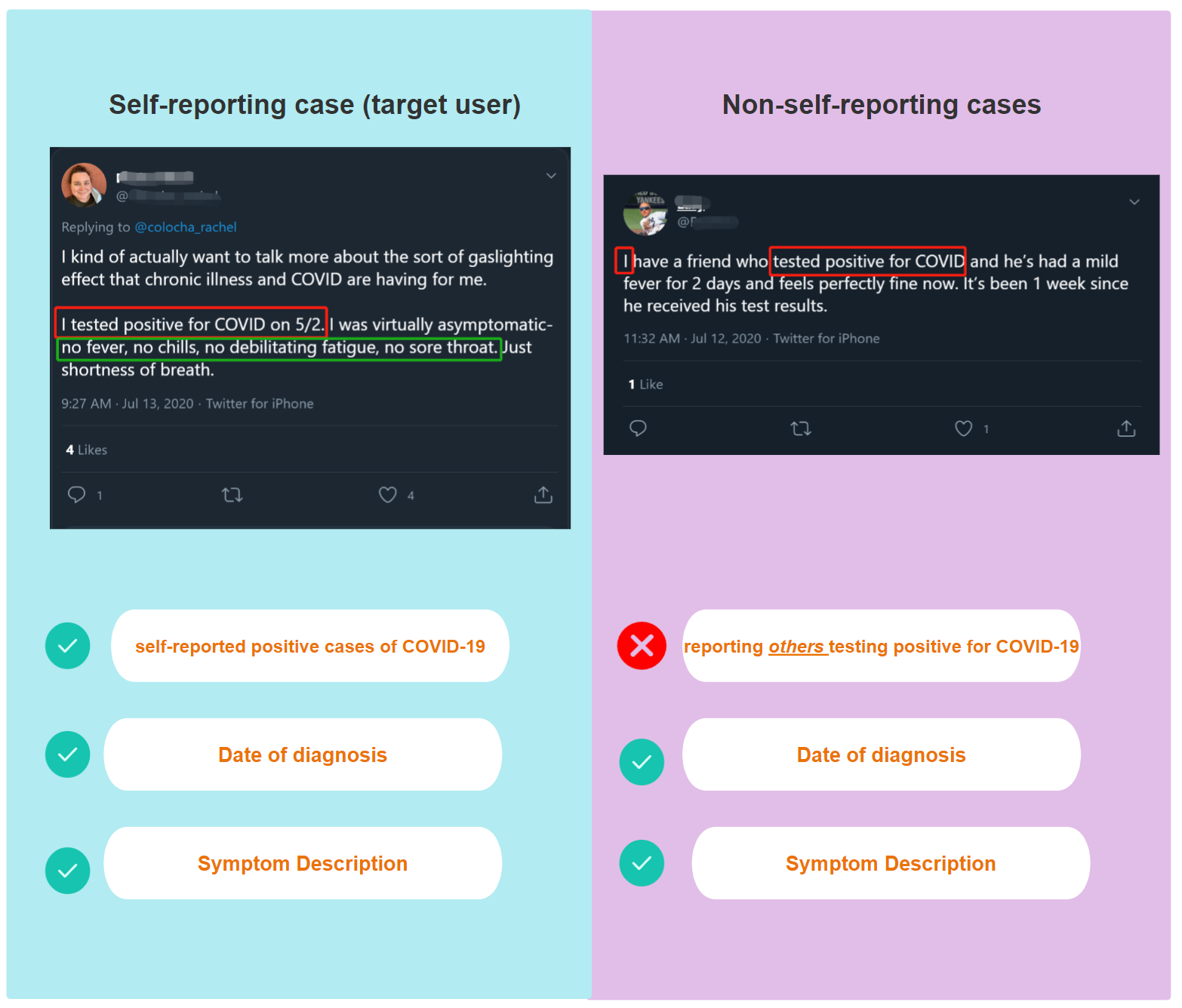


**Figure S2. An example of the target cohort**. Our target tweet on the left contains self-reported confirmed diagnosis information and associated symptom descriptions. The right is not a self-reporting tweet, although it describes the confirmed information of another person.
