# Supplementary material for: Leveraging Large Language Models for Infectious Disease Surveillance—Using a Web Service for Monitoring COVID-19 Patterns From Self-Reporting Tweets: Content Analysis
Source: J Med Internet Res. 2025 Feb 20;27:e63190. doi: 10.2196/63190 (PMC11888100; doi:10.2196/63190)
Supplement: Multimedia Appendix 6 [file jmir_v27i1e63190_app6.docx]

**Table S3. Number of parameters for conventional machine learning and large language models**

| **Models** | **Objective function** | **Number of parameters** | **Number of parameters on LoRA** |
| --- | --- | --- | --- |
| NB | Conditional likelihood | 17M | N/A |
| LR | Log-likelihood | 25M | N/A |
| SVM | Hinge loss | 43M | N/A |
| BERT | Masked Language Modeling (MLM) | 340M (frozen) | 680,000 (trainable) |
| RoBERTa | Masked Language Modeling (MLM) | 355M (frozen) | 710,000 (trainable) |
| XLNet | Permutation-based Language Modeling | 340M (frozen) | 680,000 (trainable) |
| GPT-2 | Causal Language Modeling (CLM) | 1.5B (frozen) | 3,000,000 (trainable) |
| BLOOM | Causal Language Modeling (CLM) | 560M (frozen) | 1,120,000 (trainable) |
| Llama-2 (7B) | Causal Language Modeling (CLM) | 7B (frozen) | 14,000,000 (trainable) |
