# Supplementary material for: Leveraging Large Language Models for Infectious Disease Surveillance—Using a Web Service for Monitoring COVID-19 Patterns From Self-Reporting Tweets: Content Analysis
Source: J Med Internet Res. 2025 Feb 20;27:e63190. doi: 10.2196/63190 (PMC11888100; doi:10.2196/63190)
Supplement: Multimedia Appendix 7 [file jmir_v27i1e63190_app7.docx]

**Table S4. Configuration for conventional machine learning and large language models**

| **Model** | **Text Cleaning** | **Tokenization** | **Special Tokens** | **Padding/Truncation** | **Feature Extraction** |
| --- | --- | --- | --- | --- | --- |
| **NB** | Remove noise, normalize case | Word tokenization | N/A | N/A | TF-IDF |
| **LR** | Remove irrelevant characters | Word tokenization | N/A | N/A | TF-IDF |
| **SVM** | Remove noise, normalize text | Word tokenization | N/A | N/A | TF-IDF |
| **BERT** | Lowercase, punctuation handling | WordPiece tokenizer | [CLS], [SEP] | Fixed sequence length | N/A |
| **RoBERTa** | Lowercase, punctuation handling | WordPiece tokenizer | [CLS], [SEP] | Fixed sequence length | N/A |
| **XLNet** | Lowercase, punctuation handling | SentencePiece or subword tokenization | [CLS], [SEP] | Fixed sequence length | N/A |
| **GPT-2** | Normalize case, remove unwanted characters | Byte-Pair Encoding (BPE) | endoftext token | endoftext token | N/A |
| **BLOOM** | Remove unnecessary characters | Byte-Pair Encoding (BPE) | Use tokens for sentence structure | Sequence length padding | N/A |
| **Llama-2 (7B)** | Remove unnecessary characters | Byte-Pair Encoding (BPE) | Use tokens for sentence structure | Fixed sequence length | N/A |
