# Supplementary material for: Leveraging Large Language Models for Infectious Disease Surveillance—Using a Web Service for Monitoring COVID-19 Patterns From Self-Reporting Tweets: Content Analysis
Source: J Med Internet Res. 2025 Feb 20;27:e63190. doi: 10.2196/63190 (PMC11888100; doi:10.2196/63190)
Supplement: Multimedia Appendix 8 [file jmir_v27i1e63190_app8.docx]

**Table S5. Multiple stationarity verification methods and their results**

| Methods | Results | | Predict case curve | Actual cases curve |
| --- | --- | --- | --- | --- |
| ADF | Test Statistic | | -4.66064 | -4.763675 |
|  | P-value | | 0.0001 | 0.000064 |
|  | Critical Values | 1% | -3.436 | -3.436 |
|  |  | 5% | -2.864 | -2.864 |
|  |  | 10% | -2.568 | -2.568 |
| PP | Test Statistic | | -4.642617 | -4.749968 |
|  | P-value | | 0.000874 | 0.000572 |
|  | Critical Values | 1% | -3.967 | -3.967 |
|  |  | 5% | -3.414 | -3.414 |
|  |  | 10% | -3.129 | -3.129 |
| KPSS | Test Statistic | | 0.331662 | 0.364575 |
|  | P-value | | 0.1 | 0.092424 |
|  | Critical Values | 1% | 0.739 | 0.739 |
|  |  | 5% | 0.463 | 0.463 |
|  |  | 10% | 0.347 | 0.347 |
