# Supplementary material for: Leveraging Large Language Models for Infectious Disease Surveillance—Using a Web Service for Monitoring COVID-19 Patterns From Self-Reporting Tweets: Content Analysis
Source: J Med Internet Res. 2025 Feb 20;27:e63190. doi: 10.2196/63190 (PMC11888100; doi:10.2196/63190)
Supplement: Multimedia Appendix 9 [file jmir_v27i1e63190_app9.docx]

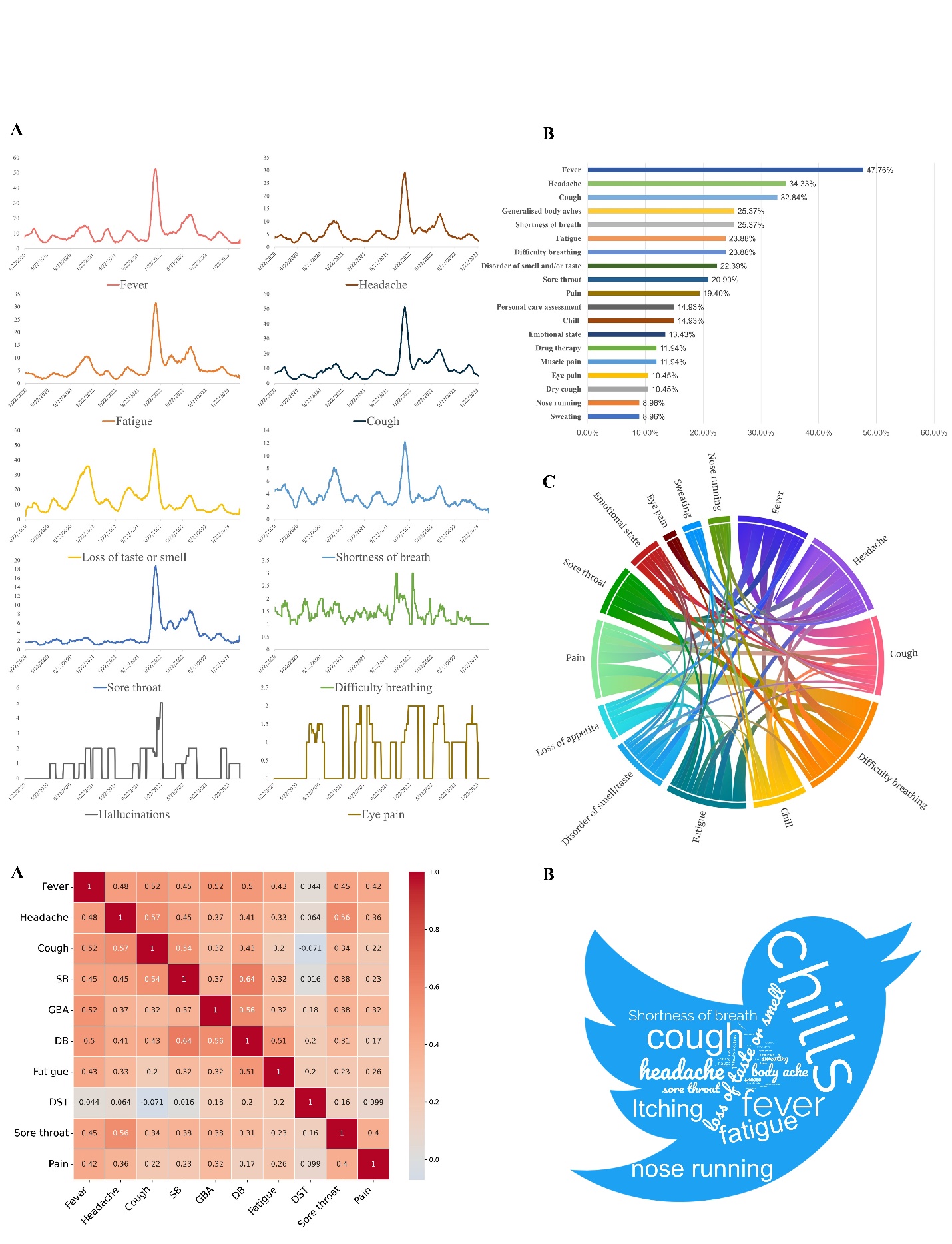


**Figure S3. Heat map of Pearson correlation coefficients among symptoms and symptoms word cloud.** (A) represents a heat map of Pearson correlation coefficients among symptoms. (B) represents the symptoms word cloud. The larger the font size, the greater the number of cases mentioning that symptom.

DB: Difficulty breathing

DST: Disorder of smell and taste

GBA: Generalized body aches

SB: Shortness of breath
