# Supplementary material for: Leveraging Large Language Models for Infectious Disease Surveillance—Using a Web Service for Monitoring COVID-19 Patterns From Self-Reporting Tweets: Content Analysis
Source: J Med Internet Res. 2025 Feb 20;27:e63190. doi: 10.2196/63190 (PMC11888100; doi:10.2196/63190)
Supplement: Multimedia Appendix 10 [file jmir_v27i1e63190_app10.docx]

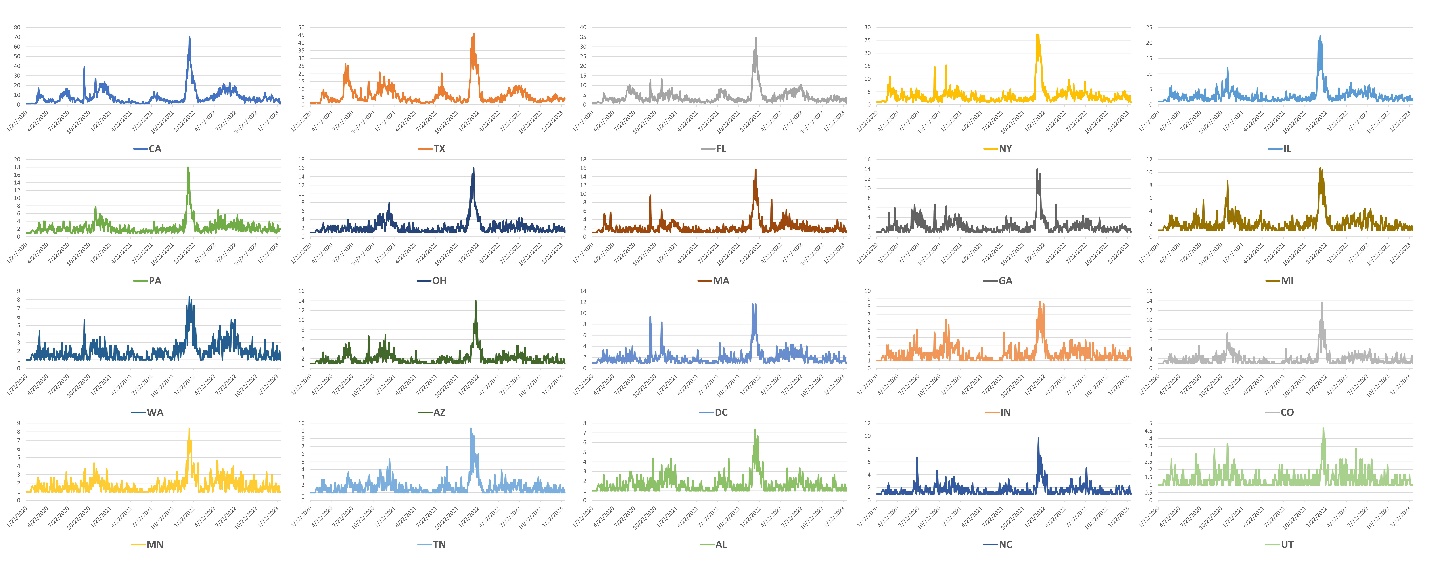


**Figure S4. Infection curve for the top 20 states with the highest infection numbers.** CA: California, TX: Texas, FL: Florida, NY: New York, IL: Illinois, PA: Pennsylvania, OH: Ohio, MA: Massachusetts, GA: Georgia, MI: Michigan, WA: Washington, AZ: Arizona, DC: District of Columbia, IN: Indiana, CO: Colorado, MN: Minnesota, TN: Tennessee, AL: Alabama, NC: North Carolina, UT: Utah
